# Phosphonothioate-Based Hydrogen Sulfide Releasing Reagents: Chemistry and Biological Applications

**DOI:** 10.3389/fphar.2017.00457

**Published:** 2017-07-10

**Authors:** Jianming Kang, Deshka L. Neill, Ming Xian

**Affiliations:** Department of Chemistry, Washington State University, PullmanWA, United States

**Keywords:** hydrogen sulfide, H_2_S, donor, phosphonothiolate, pH

## Abstract

Hydrogen sulfide (H_2_S) is a newly recognized gasotransmitter. Studies have demonstrated that the production of endogenous H_2_S and the exogenous administration of H_2_S can regulate many physiological and/or pathological processes. Therefore, H_2_S releasing agents (also known as H_2_S donors) are important research tools in advancing our understanding of the biology and clinical potential of H_2_S. Among currently available donors, GYY4137 is probably the most well-known and has been used in many studies in the past 10 years. Recently, a number of GYY4137 derivatives (e.g., phosphonothioate-based compounds) have been developed as H_2_S donors. In this review, we summarize the development and application of these donors, which include Lawesson’s reagent, substituted phosphorodithioates, cyclic phosphorane analogs, and pH-controlled phosphonamidothioates (JK donors). These donors have advantages such as good water-solubility, slow and controllable H_2_S release capability, and a variety of reported biological activities. However, it should be noted that the detailed H_2_S release profiles and byproducts under real biological systems are still unclear for many of these donors. Only after we figure out these unknowns we will see better applications of these donors in H_2_S research and therapy.

## Introduction

Hydrogen sulfide (H_2_S) has traditionally been known as a poisonous gas, with the characteristic odor of rotten eggs. However, since 2000 it has also been recognized as an important cell signaling molecule, similar to nitric oxide (NO) and carbon monoxide (CO) (Li and Moore, 2011; Olson, 2012; Wang, 2012; Kolluru et al., 2013). H_2_S is now believed to be a mediator for many physiological and/or pathological processes such as inflammation, cancer, cardiovascular diseases, and oxidative stress (Kimura and Kimura, 2004; Calvert et al., 2010; Kimura et al., 2010; King and Lefer, 2011; Whiteman et al., 2011; Köhn et al., 2012; Predmore et al., 2012; Wang, 2012; Ariyaratnam et al., 2013; Jackson-Weaver et al., 2013; Wen et al., 2013; Lee et al., 2014; Szabo et al., 2014).

**FIGURE 1:**
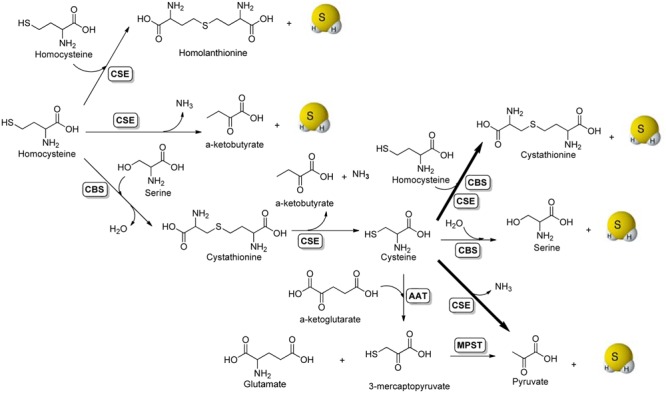
Enzymatic production of H_2_S.

The enzymatic production of H_2_S in mammalian systems has been attributed to at least three enzymes: cystathionine β-synthase (CBS), cystathionine γ-lyase (CSE), and 3-mercaptopyruvate sulfurtransferase (MPST) (Hu et al., 2011; Paul and Snyder, 2012; King, 2013; Banerjee, 2017). It is believed that CBS is the main H_2_S synthase in the nervous system, whereas CSE plays the same role in most peripheral tissues, except for the liver and kidney, which contain both enzymes in substantial amounts. Enzyme-mediated H_2_S production from sulfur-containing amino acids is summarized in **Figure 1**. Briefly, the condensation of two molecules of homocysteine by CSE generates homolanthionine and H_2_S. CSE can also catalyze the α-γ-elimination of homocysteine to produce α-ketobutyrate, ammonia, and H_2_S. CBS catalyzes the β-replacement of serine by homocysteine to form cystathionine, which can be further converted to cysteine, α-ketobutyrate and ammonia by CSE. With cysteine as the key intermediate, both CBS and CSE can produce H_2_S via β-replacement by homocysteine. In addition, CBS can swap cysteine for serine eliminating H_2_S in the presence of water. Another route of CSE-catalyzed H_2_S generation is via the α, β elimination of cysteine, to form pyruvate and ammonia. This is the major pathway for H_2_S formation by CSE under physiologically relevant substrate concentrations. For CBS-catalyzed H_2_S production, the β-replacement of cysteine by homocysteine is the dominant route. The third main pathway for H_2_S production requires two enzymes, aspartate aminotransferase (AAT) and MPST. This pathway operates mainly in mitochondria (Whiteman et al., 2011). AAT catalyzes the transamination reaction between cysteine and α-ketoglutarate to produce 3-mercaptopyruvate and glutamate, which in a subsequent step catalyzed by MPST liberates H_2_S and pyruvate. In addition to these enzymatic pathways, H_2_S can also be generated endogenously through the non-enzymatic reduction of elemental sulfur by reducing equivalents supplied through the glycolytic pathway (Kolluru et al., 2013).

The biological functions of H_2_S may result from its reactions with biomolecules under physiological or pathological conditions. One of the most important molecular mechanisms of H_2_S signaling is *S*-persulfidation, e.g., converting protein’s thiols (–SH) to persulfides (–SSH) (Pan and Carroll, 2013; Paulsen and Carroll, 2013; Zhang et al., 2014). Many protein targets of *S*-persulfidation, including receptors, ion channels and enzymes, have been identified. This process is believed to be important as it provides a possible route by which H_2_S alters the functions of a wider range of cellular proteins (Paul and Snyder, 2012; Vandiver et al., 2013; Yang et al., 2013). As for the mechanisms behind persulfidation, it is believed that H_2_S can easily react with oxidized thiols such as sulfenic acids (-SOH) or nitrosothiols (-SNO) to form persulfides (-SSH). It is also believed that H_2_S can react with disulfides to form persulfides. However, the reactions of H_2_S with disulfides, in particularly low molecular weight disulfides, are slow (Liu and Chang, 1987; Francoleon et al., 2011; Cuevasanta et al., 2015; Vasas et al., 2015). Protein environment may significantly enhance the reaction. For example, the reaction of H_2_S with an active site disulfide in SQR is accelerated by ∼10^6^-fold with respect to free cystine (Jackson et al., 2012; Libiad et al., 2014; Mishanina et al., 2015). The concentration of disulfides in cytosol is usually low. Therefore the reaction between disulfides and H_2_S is likely to be more significant in locations like endoplasmic reticulum or under oxidative conditions. Recent studies suggest that H_2_S may interact with NO and related species. H_2_S reacts with NO to form thionitrous acid (HSNO), which is the smallest *S*-nitrosothiol, whose metabolites, including NO, NO^-^, and NO^++^, have significant physiological functions (Filipovic et al., 2012b). H_2_S can also act as a reductant and react with a number of one-electron and two-electron oxidants, such as hydroxyl radical (HO^∙^), nitrogen dioxide (NO_2_), superoxide ($O_{2}^{•–}$), peroxynitrite (ONOO^-^), and hydrogen peroxide (H_2_O_2_) (Carballal et al., 2011; Filipovic et al., 2012a). Removal of these reactive oxygen species reduces intracellular redox imbalance, which is involved in the pathophysiology of a wide array of human diseases. Moreover, H_2_S can easily react with metalloproteins, particularly heme-containing ones. The smaller size of H_2_S compared to other low molecular weight thiols would make it more accessible to metal centers. Binding of H_2_S to cytochrome c oxidase at moderately high H_2_S concentrations is associated with the induction of the suspended animation state (Blackstone et al., 2005). Although H_2_S inhibits cytochrome c oxidase, the situation is more complicated in tissues. At high concentrations, the mitochondria respiratory chain is inhibited. At low concentrations, H_2_S stimulates oxygen consumption (Cooper and Brown, 2008). In addition, H_2_S binds to hemoglobin forming ferric heme, which may be reduced by a second mole of H_2_S to generate ferrous heme and hydrogen persulfide (Pietri et al., 2009). These intermediates may also be involved in some physiological/protective effects.

In the exploration of H_2_S biology it is also important to explore the understudied chemistry and reactivity of H_2_S, and to be aware of the problems associated with the choice of resources used to produce H_2_S in *in vitro* and *in vivo* experiments. So far, one of the main challenges in the H_2_S field is precise delivery of H_2_S. Moreover, the ideal concentrations of H_2_S in physiology and in therapy need to be better understood, because the therapeutic windows in almost all known pharmacological experiments for H_2_S are very narrow. H_2_S gas is the authentic resource with a volatile nature and unpleasant odor. Although H_2_S gas has been used in many experiments, showing promising biological effects, it is far from ideal due to the difficulties in obtaining and maintaining constant concentrations, as well as the possible toxic effects of H_2_S in excess (Blackstone et al., 2005; Blackstone and Roth, 2007; Collman et al., 2009; Xue et al., 2013). Inorganic sulfide salts, such as sodium sulfide (Na_2_S) and sodium hydrogen sulfide (NaHS), are also widely used H_2_S equivalents (Zhao et al., 2001; Zanardo et al., 2006; Zhang et al., 2013). These sulfide salts conveniently generate H_2_S, and no byproducts are produced after H_2_S generation. However, these compounds are short-lived and un-controlled H_2_S donors, as they release H_2_S immediately once their solution is prepared. In addition, H_2_S is very volatile in aqueous solution. DeLeon et al. (2012) have demonstrated that the half-life of 10 μM Na_2_S solution is 0.5 min and no detectable H_2_S is left after 12 h. This uncontrollable and rapid release results in a spike of H_2_S concentration that can cause severe damages *in vivo*. It should also be noted that commercial sulfide salts usually have different levels of impurities, which could affect experimental outcomes. With all these concerns, caution should be taken when using sulfide salts as H_2_S donors.

Given the problems of H_2_S gas and sulfide salts, synthetic and so-called ‘controllable’ H_2_S donors have received considerable attention in recent years. So far, a number of donors have been developed and their H_2_S releases are triggered by different mechanisms, such as hydrolysis, thiol activation, and photolysis (Li et al., 2008; Zhao et al., 2011, 2013, 2014, 2015; Devarie-Baez et al., 2013; Fukushima et al., 2014; Zheng et al., 2017). Among all these synthetic donors, GYY4137 is the most widely used one and a variety of biological activities have been reported (Li et al., 2008). The core structure of GYY4137, i.e., phosphonothioate, represents a valuable template for the design of H_2_S donors. In this review, we summarize the results of all known phosphonothioate-based H_2_S donors in the context of their chemistry and biological activities.

## Lawesson’S Reagent

**FIGURE 2:**
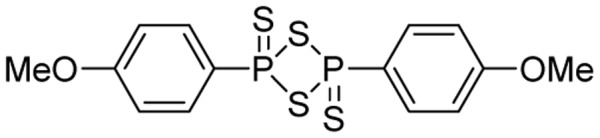
Lawesson’s reagent.

Lawesson’s reagent (2,4-bis(4-methoxyphenyl)-1,3,2,4-dithiadiphosphetane-2,4-disulfide, **Figure 2**) is a sulfurization reagent often seen in organic synthesis (Ozturk et al., 2007). Its biological activity was largely unknown. With the increasing interest in H_2_S biology, Lawesson’s reagent was considered as an H_2_S donor and some interesting activities have been reported. In 2009, Lawesson’s reagent was used as an H_2_S equivalent to study the effects of H_2_S on the inflammation and ulceration of the colon in a rat model of colitis (Wallace et al., 2009). Treatment with Lawesson’s reagent and the standard NaHS resulted in a significant reduction in the severity of colitis, as measured by a decrease in colon thickness and reduced TNF-α mRNA expression, a proinflammatory cytokine. Also Medeiros et al. (2009) found that Lawesson’s reagent could prevent ethanol-induced gastric damage in a dose-dependent manner and the primary mechanism was believed to be the activation of K_ATP_ channels and afferent neurons/TRPV1 receptors. Nicolau et al. (2013) reported that Lawesson’s reagent exhibited H_2_S-relavent protective effects on gastric damage, induced by alendronate (ALD)-a bisphosphonate drug for the prevention and treatment of bone diseases. It was also found that the blockade of K_ATP_ channels alone reversed Lawesson’s reagent’s protective effects against ALD-induced gastric damage, suggesting an involvement of K_ATP_ channels in H_2_S gastroprotective effects. It should be noted that the H_2_S releasing mechanism of Lawesson’s reagent is thought to be due to spontaneous hydrolysis. However, the hydrolysis kinetics and by-products are still unclear. Moreover, the poor solubility of Lawesson’s reagent in physiological solutions limits its applications.

## GYY4137

**FIGURE 3:**
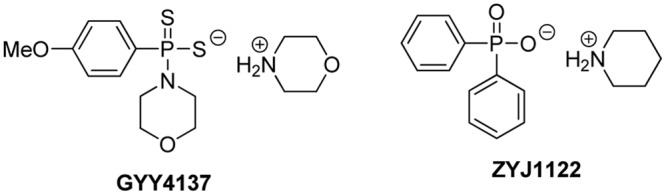
The structure of GYY4137 and its control compound.

GYY4137, or morpholin-4-ium 4 methoxyphenyl(morpholino) phosphinodithioate, is a Lawesson’s reagent derivative and can be easily prepared by reacting Lawesson’s reagent with morpholine (Li et al., 2008) (**Figure 3**). GYY4137 is one of the first organic small molecule H_2_S donors reported. This compound has excellent water solubility and is believed to release H_2_S very slowly under physiological conditions. H_2_S release from GYY4137 was also found to be pH and temperature dependent, with a less release at lower temperatures like 4°C and a greater release at acidic pH like pH 3.0 (Li et al., 2008).

Unlike commonly used sulfide salts such as Na_2_S and NaHS, which release H_2_S instantaneously upon dissolving in aqueous solutions, H_2_S release from GYY4137 is at a sustained rate and remains at a low level, even after several days (measured in buffers) (Li et al., 2008). When administrated into animals, GYY4137 can cause and maintain elevated H_2_S levels for a long period of time. For example, after intravenous or intraperitoneal administration of GYY4137 (133 μmol/kg) to Sprague-Dawley rats, plasma H_2_S concentration was increased at 30 min and remained elevated over the 180-min time course.

GYY4137 is a very popular H_2_S donor and many researchers have used it to study the functions of H_2_S. A variety of H_2_S-relavent activities have been reported. In the original paper that reported the discovery of GYY4137 (Li et al., 2008), it was found that isolated blood vessels respond to the presence of GYY4137 (200 μM) with a slowly developing (∼10 min) but sustained (∼40 min) vasorelaxation while NaHS (300 μM) caused rapid, transient, and reversible (∼20 to 30 s) relaxation of aortic rings. GYY4137 (26.6–133 μmol/kg) showed a slow (at 30 min) drop in blood pressure while NaHS (2.5–20 μmol/kg) caused fast (10–30 s) and dose-related decrease in blood pressure. GYY4137 has also been shown to exert cytoprotective effects under different pathological conditions. In a report by Fox et al. (2012) GYY4137 (10–200 μM) treatment significantly inhibited oxidative stress-induced mitochondrial dysfunction and cell death through pathways involving Akt/PI3K-dependent signaling in human mesenchymal progenitor cells and human articular chondrocytes. In contrast, cell death increased with the pharmacological inhibition of H_2_S synthesis or by CBS/CSE-siRNA treatment. In another study, Wei et al. (2014) demonstrated that GYY4137 (100 and 200 μM) protects against high glucose-induced cytotoxicity by activation of the AMPK/mTOR signaling pathway in H9c2 myocardial cells. *In vivo* cytoprotection was carried out in a hyperoxia-induced lung injury model in new born rat pups by Vadivel et al. (2014). Intraperitoneal administration of GYY4137 (37.75 mg/kg/day) preserved and restored normal alveolar growth, attenuated pulmonary hypertension, and prevented pulmonary artery smooth muscle cell proliferation in rat pups exposed to hyperoxia.

GYY4137 has also been used in cellular and *in vivo* models to study H_2_S’s anti-inflammatory effects. Whiteman et al. (2010) showed that in murine RAW 264.7 macrophages, pretreatment with GYY4137 (50–200 μM) significantly and concentration-dependently inhibits LPS-induced release of pro-inflammatory mediators such as IL-1b, IL-6, TNF-α, NO, and PGE2. It also increased the synthesis of the anti-inflammatory chemokine IL-10 through NF-κB/ATF-2/HSP-27-dependent pathways. In contrast, NaHS exhibited a biphasic effect on pro-inflammatory mediators and, at high concentrations (>200 μM), increased the synthesis of pro-inflammatory mediators. Burguera et al. (2014) evaluated the effects of NaHS and GYY4137 on inflammation in articular chondrocytes. Both NaHS and GYY4137 led to significantly reduced NO, PGE-2, IL-6 and MMP13, which was achieved by downregulation of NOS2, cyclooxigenase-2 (COX2), prostaglandin E synthase (PTGES), IL-6 and MMP13 through NF-κB inhibition. GYY4137 has also been shown to be anti-inflammatory *in vivo*. In a report by Li et al. (2009), the intraperitoneal administration of GYY4137 (50 mg/kg) to conscious rats after LPS decreased the subsequent increase in plasma pro-inflammatory cytokines (TNF-α, IL-1β, IL-6) via the suppression of NF-κB activity. GYY4137 administration also decreased the LPS-induced increase in lung myeloperoxidase activity, elevated anti-inflammatory cytokine IL-10 concentration in plasma, and reduced tissue inflammation, although the mechanism is still not clear. Wu et al. (2015) showed that in Coxsackie virus B3 (CVB3)-infected rat cardiomyocytes, GYY4137 suppressed CVB3-induced secretion of LDH, CK-MB and pro-inflammatory cytokines, such as tumor necrosis factor-α, interleukin (IL)-1β and IL-6. They hypothesized that the mechanism may be associated with the suppression of NF-κB and MAPK signaling pathway activation.

It was proposed that low (endogenous) H_2_S concentrations tend to promote, while high (exogenous) H_2_S concentrations tend to inhibit cancer cell proliferation (Hellmich and Szabo, 2015). The anti-cancer activity of GYY4137 was first reported by Lee et al. (2011) in both *in vitro* and *in vivo* models. In this study, GYY4137 (100 μM to 1 mM) caused concentration-dependent G_2_/M phase cell cycle arrest and PARP-/caspase-9-dependent apoptosis of seven different human cancer cell lines (HeLa, HCT-116, HepG2, HL-60, MCF-7, MV4-11 and U2OS) but did not affect normal human lung fibroblasts (IMR90, WI-38) suggesting that cancer cells can be killed selectively. Interestingly NaHS was less potent and not active, and ZYJ1122 (**Figure 3**), a sulfur lacking control compound, was also inactive. These control results suggest that the anti-cancer effects were due to sustained exposure to low levels of H_2_S. For *in vivo* experiments, the intraperitoneal administration of GYY4137 (100–300 mg/kg/day) significantly reduced tumor growth in HL-60 and MV4-11. Lu et al. (2014) reported that GYY4137 (10–50 mg/kg) significantly inhibited tumor growth in the subcutaneous HepG2 xenograft mice model, possibly by suppression of STAT3 activation. Recently GYY4137 was found to show interesting activity against virus infection for respiratory syncytial virus, human metapneumovirus, and Nipah virus (Li et al., 2015). GYY4137 treatment showed no effect on viral genome replication or viral mRNA/protein synthesis. However, it inhibits syncytium formation and virus assembly/release. In this study, the concentrations of GYY4137 applied were quite high (up to 10 mM).

So far GYY4137 has been widely used in H_2_S research and is now considered a standard H_2_S donor. However, caution should be taken in choosing GYY4137 in studies. Normally, H_2_S levels before and after GYY4137 administration should be validated. Due to its fixed and very slow H_2_S release capability, high concentrations (up to mM levels) are often required to accumulate enough H_2_S for biological studies. Such concentrations are not therapeutically preferred. In addition, the mechanism of H_2_S release from GYY4137 is still unclear. It was proposed to be hydrolysis based, but the kinetics and decomposition intermediate/final product have not been well characterized. In biological systems, GYY4137 may interact with certain biomolecules to facilitate H_2_S release while the identity of those molecules is unclear. In this regard, appropriate control experiments should be carried out. Recently ZYJ1122 was used as a control compound. However, given its different structure from GYY4137, whether or not it is a suitable control compound is still uncertain.

## GYY4137 Analogs

**FIGURE 4:**
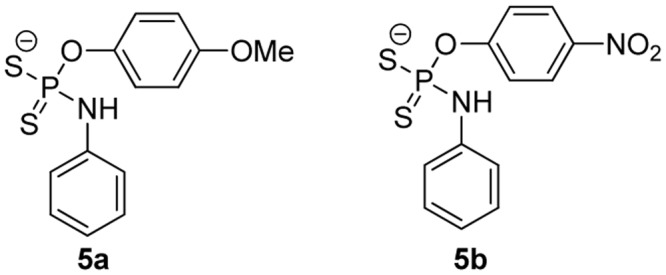
*O*-Substituted phosphorodithioates.

Given the fixed H_2_S-release capability of GYY4137, researchers have tried to modify its structure with the hope of changing the donor’s H_2_S release profile. Park et al. (2013) prepared a series of *O*-aryl- and alkyl-substituted phosphorodithioates by replacing the phenyl-phosphorus linkage of GYY4137 with an oxygen-phosphorus bond (such as **5a, 5b, Figure 4**). It was found that *O*-aryl-substituted analogs showed similar slow and sustainable H_2_S generation as GYY4137, while *O*-alkyl-substituted analogs only showed trace amount of H_2_S release. It was hypothesized that *O*-alkyl substitutions increased the stability of phosphorodithioates and therefore decreased the efficiency of hydrolysis to produce H_2_S. These donors showed some protective activity against H_2_O_2_-induced oxidative damage in H9c2 cells as well.

**FIGURE 5:**
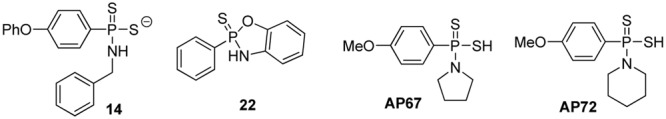
Structures of representative GYY4137 analogs.

Feng et al. (2015) synthesized a series of GYY4137 analogs with different H_2_S releasing rates, and tested their anti-proliferative activity against several solid tumor cell lines. Two representative donors **14** and **22** are shown in **Figure 5**. To validate H_2_S production from the donors, confocal imaging using a fluorescent probe was employed to quantify intracellular H_2_S. Intracellular pH (pHi) was also used as an alternative indicator for intracellular H_2_S, because a decreased pHi should be due to increased glycolysis, causing overproduction of lactic acid and decreased anion and Na/H exchange activity, thus introducing intracellular acidification. At 50 μM, **14** reduced pHi to 6.75 while GYY4137 reduced pHi to 7.14, indicating that **14** released more H_2_S than GYY4137. Confocal imaging also confirmed better H_2_S releasing ability of **14** compared to GYY4137. At 200 μM, **14** released 41.5 μM H_2_S in MCF7 cells at 6 h but GYY4137 released only 5.41 μM. Greater cell permeability of **14** was believed to be a key factor for its improved intracellular H_2_S releasing ability. The authors also explored a series of 2,3-dihydro-2-phenyl-2-sulfanylenebenzo[d][1,3,2]oxazaphospholes by cyclizing these initial structures. They proposed that cyclization may lead to more control over the reactivity due to limited conformational freedom. Compound **22** was shown to have the best H_2_S releasing capacity and **22** was also the most potent compound against MCF7 (IC_50_ = 5.7 μM) and SKOV3 (IC_50_ = 6.12 μM) cell lines. From all compounds studied in this work, **22** was the safest compound with an improved therapeutic window of over eightfold in WI38 (IC_50_ > 50 μM) cells. At 100 μM, **22** inhibited MCF7 spheroid growth by nearly 70% after 14 days which further characterized its anticancer activity. Mechanism study showed an increase in cleaved poly (ADP-ribose) polymerase (PARP) and activated caspase-7 in MCF7 cells, indicating that the mechanism of cancer cell death was apoptosis.

Protonated GYY4137 analogs such as AP67 and AP72 have also been prepared and studied (Chitnis et al., 2013). These compounds likely undergo ionization to form their corresponding salts under physiological pH. AP67 and AP72 have shown potent vasodilatory effects on pre-contracted bovine posterior ciliary arteries (PCAs) through endogenous NO synthesis and the activation of K_ATP_ channels. Further activities for these interesting donors are still to be discovered.

## pH-Dependent Phosphonamidothioate-Based Donors (JK Donors)

H_2_S release from GYY4137 and related phosphonothioate donors is suggested to be hydrolysis based. However, these donors’ H_2_S production in aqueous buffers was found to be very slow, suggesting that the intermolecular hydrolysis reaction with water is quite slow. While this very slow H_2_S release is not necessarily a problem, it would be ideal to also have access to faster release donors, so that researchers would have more options. With this idea in mind, Kang et al. (2016) reported a series of phosphonamidothioate-based donors (e.g., JK donors), which showed enhanced H_2_S releasing capabilities.

**FIGURE 6:**
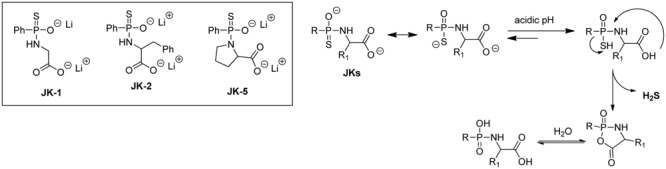
The structures of selected JK donors and the H_2_S release mechanism.

As shown in **Figure 6**, these donors contain a free carboxylate group adjacent to the phosphonamidothioate core. Under neutral or slightly acidic pH, JKs are expected to be protonated to form phosphorothioates. Then, the nucleophilic carboxylate can attack the phosphorothioate center to push H_2_S away and form a five-membered-ring intermediate (or its hydrolyzed product). This cyclization process is believed to contribute to the enhancement of H_2_S release. In this study, the release of H_2_S from these donors was tested using a modified zinc sulfide-precipitation-based methylene blue (MB) method, because the standard MB method utilizes strongly acidic conditions which can cause false signals. As expected, JK donors showed a more enhanced H_2_S release than GYY4137. Most interestingly, the H_2_S release from JKs was found to be very pH-dependent, with a faster and more H_2_S release under acidic pH and a slower and less abundant H_2_S release at neutral or basic pH. Moreover, structural modifications could also affect H_2_S release. For example, JK-1 releases barely detectable H_2_S at pH 7.4 and 8, but significant amounts of H_2_S at pH 5 or 6. The introduction of a benzyl group at the α-position makes the donor, JK-2, release obviously detectable H_2_S even under neutral or weak basic pH. JK-5, on the other hand, showed no H_2_S release at all, presumably the rigid proline ring inhibits intramolecular cyclization.

A pH decrease under pathological conditions, such as cancer and ischemia/reperfusion injuries are known. As such, JK donors are expected to be useful tools for the study of H_2_S and its role under those pathological conditions. Indeed, the cytoprotective effects of JK-1 and JK-2 on a cellular model of myocardial ischemia/reperfusion (MI/R) injury were demonstrated. These donors also showed significant protection in a murine model of MI/R injury (induced by subjecting mice to 45 min of left ventricular ischemia, followed by 24 h of reperfusion). Compared to vehicle-treated mice, mice treated with donors showed significantly reduced infarct size per area-at-risk (INF/AAR). The protective effects were also validated by measuring circulating cardiac troponin I levels, the marker for acute myocardial infarction. Troponin I levels were significantly reduced with JK-1 or JK-2 treatment.

Recently, some other H_2_S-relevant activities of JK donors have been revealed. In one example, Wu et al. (2016) developed a JK-1 based nanofiber, PCL-JK1, through electrospinning of polycaprolactone (PCL) containing JK-1. Compared to JK-1, PCL-JK1 showed a similar pH regulated H_2_S releasing behavior. However, H_2_S release was prolonged by the fibrous matrix as demonstrated by slower releasing rates. Cell-compatibility was carried out using NIH 3T3 fibroblast cells and no cytotoxicity was observed at both pH 6.0 and pH 7.4 upon culturing for 72 h. The use of PCL-JK1 as a wound dressing agent toward a cutaneous wound model was tested. PCL-JK1 was found to significantly enhance the wound repair and regeneration efficiency compared with PCL fiber alone, likely due to the ability of H_2_S to inhibit inflammation, reduce oxidative damage and increase angiogenesis. In another example, Yang et al. (2017) demonstrated that the intragastrical (IG) pre-administration of JK-1 protects gastric mucosa from aspirin (ASP)-induced gastric mucosal injury *in vivo*. Preconditioning with JK-1 alleviated ASP-induced inflammation response which was further validated by decreased levels of pro-inflammatory factors IL-6 and TNF-α. JK-1 was also shown to blunt ASP-induced oxidative stress evidenced by reduced MPO levels in gastric tissues. *In vitro* cellular experiments suggested that the exposure of gastric mucosal epithelial (GES-1) cells to HClO, imitating MPO-driven oxidative injury, decreased cell viability, increased apoptotic rate and damaged mitochondrial function, which were reversed by pretreatment with JK-1.

## Conclusion

GYY4137 and related analogs have been widely used in H_2_S studies due to their excellent solubility and slow H_2_S release capability. A summary of key information of some representative donors in this category is shown in **Table 1**. Current results suggest the phosphorothioate structure is a valuable template for the design of H_2_S donors. Structural modifications on the core structure can lead to donors with varied and controllable H_2_S release ability. It should be noted that their H_2_S release profiles in simple systems such as aqueous buffers are usually available. However, information of their release (kinetics, mechanism, intermediates, byproducts, etc.) in real biological systems (cells, tissues, etc.) is difficult to obtain. This lack of information can be a problem. In some cases it is uncertain if the observed activities are due to H_2_S, or the donor molecule itself, or the decomposed by-product(s). Phosphorodithioates are likely to be biologically active molecules. In order to use GYY4137 type molecules as H_2_S donors, one has to clearly understand the mechanism and profiles of H_2_S release from these molecules, as well as the identity of the by-products. Only with this knowledge available can appropriate control experiments be designed and carried out.

**Table 1:** Key information of representative phosphonothioate-type donors.

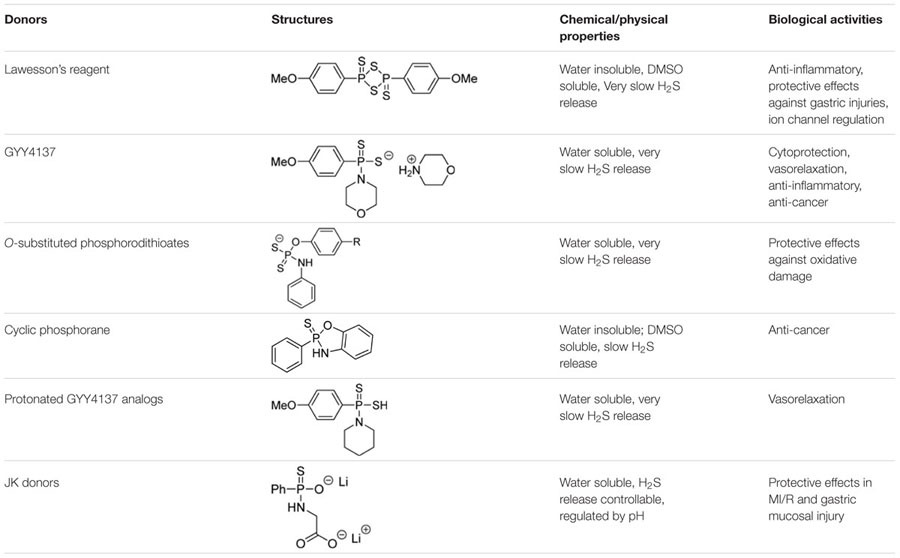

## Author Contributions

JK and DN collected and analyzed references. JK, DN, and MX wrote the article. All authors listed have made substantial contribution to the work and approved it for publication.

## Conflict of Interest Statement

The authors declare that the research was conducted in the absence of any commercial or financial relationships that could be construed as a potential conflict of interest.
